# Systemic sclerosis and tachycardia–bradycardia syndrome: a case report

**DOI:** 10.1186/s13256-022-03462-z

**Published:** 2022-06-21

**Authors:** Raid Faraj, Nabil Laktib, Safae Hilal, Fadoun Hassan, Amine Krimech, Alaa Bouanani, Mohamed Sarsari, Ibtissam Fellat, Jamila Zarzur, Mohamed Cherti

**Affiliations:** Mohammed V University of Rabat, Rabat, Morocco

**Keywords:** Systemic sclerosis, Tachycardia–bradycardia syndrome, Sick sinus syndrome, 24-hour ambulatory ECG, Case report

## Abstract

**Background:**

Systemic sclerosis is a multisystemic character autoimmune disease. It is characterized by vascular dysfunction and progressive fibrosis affecting mainly the skin but also different internal organs. All heart structures are commonly affected, including the pericardium, myocardium, and conduction system. However, tachycardia–bradycardia syndrome is not common in the literature as a cardiac complication of systemic sclerosis.

Case presentation

We report a case of tachycardia–bradycardia syndrome in a 46-year-old Moroccan woman followed for systemic sclerosis with cutaneous, vascular, and articular manifestations. The diagnosis was based mainly on patient-reported symptoms and electrocardiogram data. A permanent pacemaker was implanted, allowing the introduction of beta-blockers with good outcomes.

**Conclusions:**

This case aims to show that even minor electrocardiogram abnormalities should be monitored in this group of patients, preferably by 24-hour ambulatory electrocardiogram because they could be a good indicator of the activity and progression of cardiac fibrosis.

## Background

Owing to the differences in disease classification, the prevalence and incidence rates of systemic sclerosis (SSc) vary widely. Up to 56 new cases per million persons are reported each year, mostly females [1]. On the basis of the extent of cutaneous involvement and the accompanying pattern of internal organ involvement, SSc could be classified as limited cutaneous SSc, diffuse cutaneous SSc, SSc sine scleroderma, and SSc overlap syndrome [2]. Cardiac involvement is common in patients with SSc. It includes myocardial dysfunction, coronary artery disease, pericardial disease, valvular diseases, and arrhythmias. Even if it is commonly clinically occult, cardiac involvement in this population highly affects the prognosis of SSc and represents a major cause of mortality [3]. Sick sinus syndrome (SSS) is a sinoatrial dysfunction mostly related to sinoatrial node and surrounding atrial myocardium senescence. The combination of atrial tachyarrhythmias and atrioventricular nodal conduction disturbances defines tachycardia–bradycardia syndrome.

We were not able to find a case of scleroderma associated with tachycardia–bradycardia syndrome; however, sick sinus syndrome was found by electrophysiologic examination in some systemic sclerosis cases associated with cardiac involvement [4]. To the best of our knowledge, this is the first published case of tachycardia–bradycardia syndrome secondary to SSc.

Our case report was written according to the CARE guidelines [5].

## Case presentation

We report the case of a 46-year-old Moroccan female admitted to the cardiology B department of Ibn Sina University Hospital, presenting asthenia and several presyncope episodes preceded by palpitations attacks 2 months earlier. She had a history of SSc evolving for 18 years with diffuse cutaneous, articular, and vascular involvement (Raynaud phenomenon) treated with methotrexate, long-acting dihydropyridine calcium channel blockers, and low-dose glucocorticoids.

**Fig. 1:**
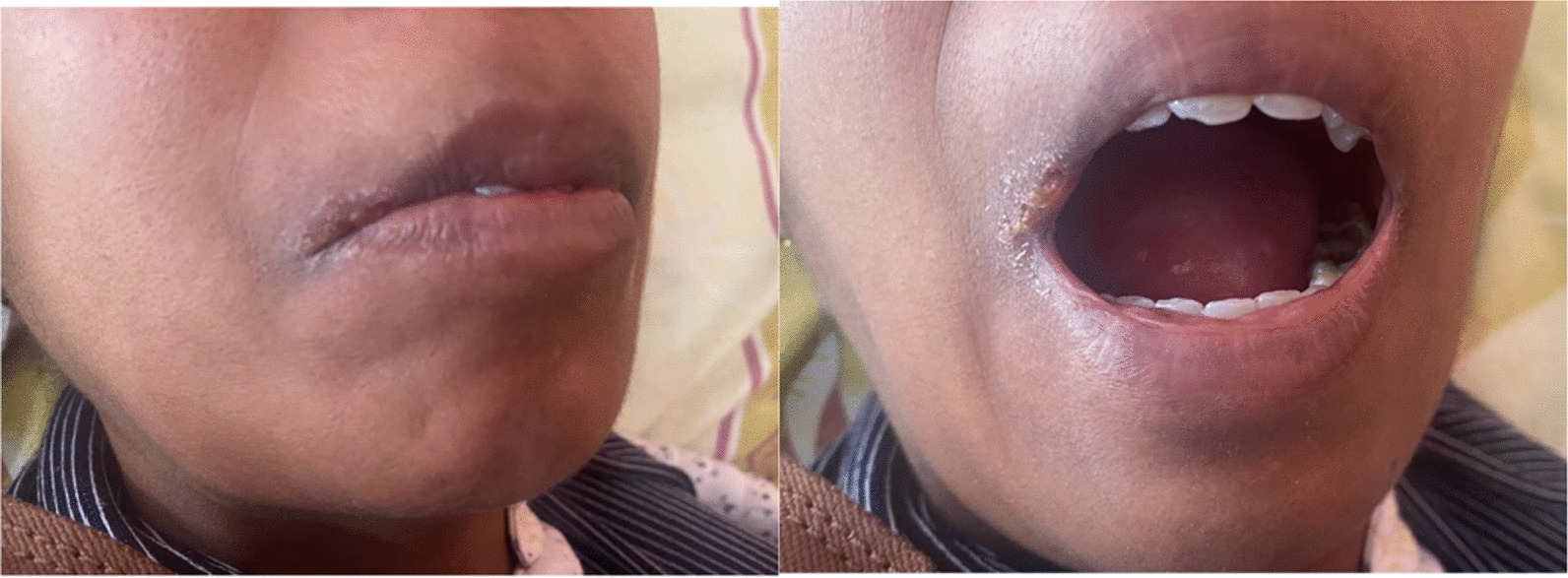
Orofacial appearance in systemic sclerosis: **A** taut facial skin, loss of wrinkles and skin folds, puckered mouth, and **B** restricted mouth opening with labial erosions

**Fig. 2:**
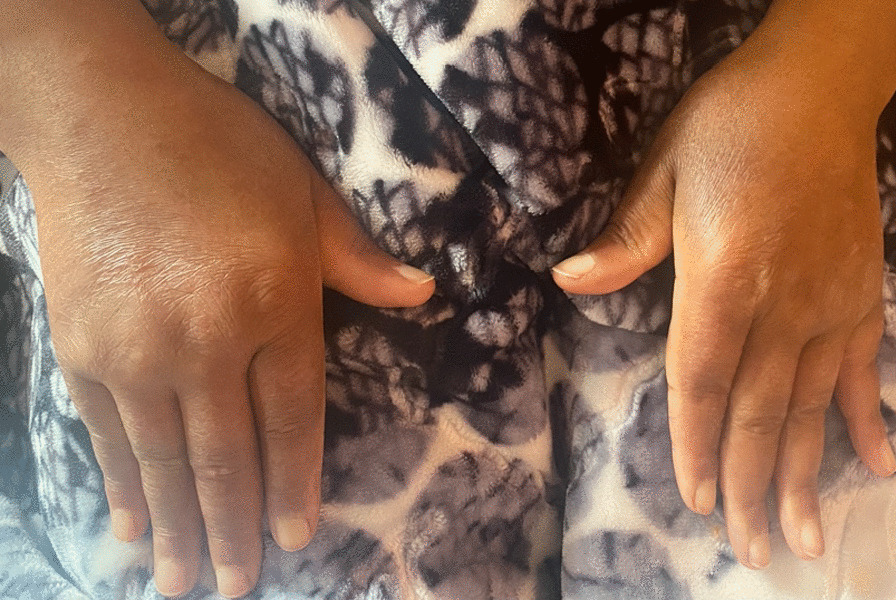
Sclerodactyly, tightening, and thickening of the skin on the fingers and hands

The patient’s blood pressure was 150/85 mm Hg, her heart rate was 42 beats per minute, and her respiratory rate was 16 breaths per minute. Physical examination revealed Mauskopf facies (Fig. 1A): expressionless face, shiny skin, pinched nose, thinning of lips with small oral aperture associated with labial erosions (Fig. 1B). In addition, sclerodactyly (Fig. 2) and prayer sign were noted. The modified Rodnan skin score was 23 (total score 0–51).

**Fig. 3:**
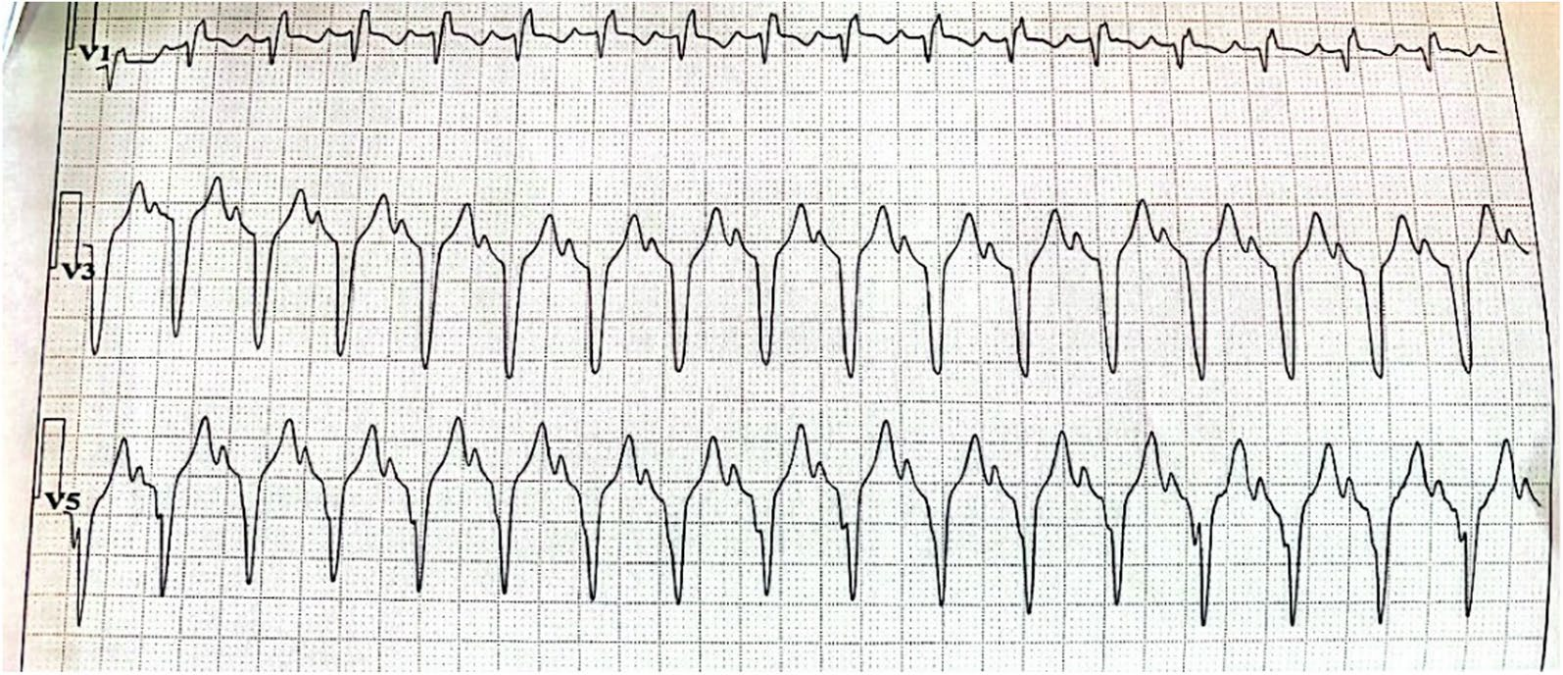
Holter ECG showing supraventricular tachycardia (SVT) with a wide QRS complex due to a right bundle branch block (RBBB)

A Holter electrocardiogram (ECG) performed 3 weeks before her admission revealed the presence of salvos of supraventricular extrasystoles and multiple supraventricular tachycardia episodes without any conduction disorder (Fig. 3).

**Fig. 4:**
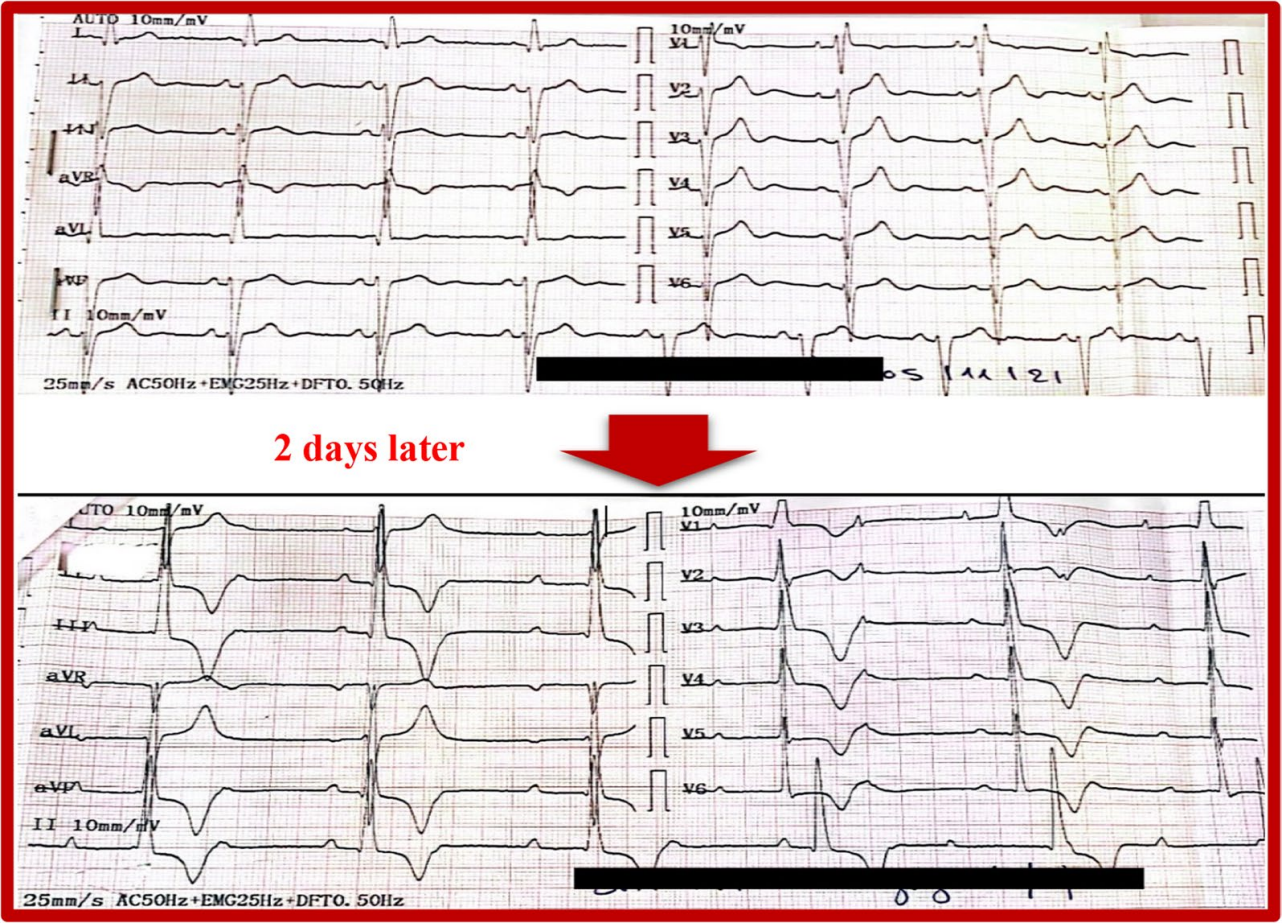
**A** Bifascicular block combining a right bundle branch block (RBBB) with a left anterior hemiblock (LAHB). **B** Complete atrioventricular block

**Fig. 5:**
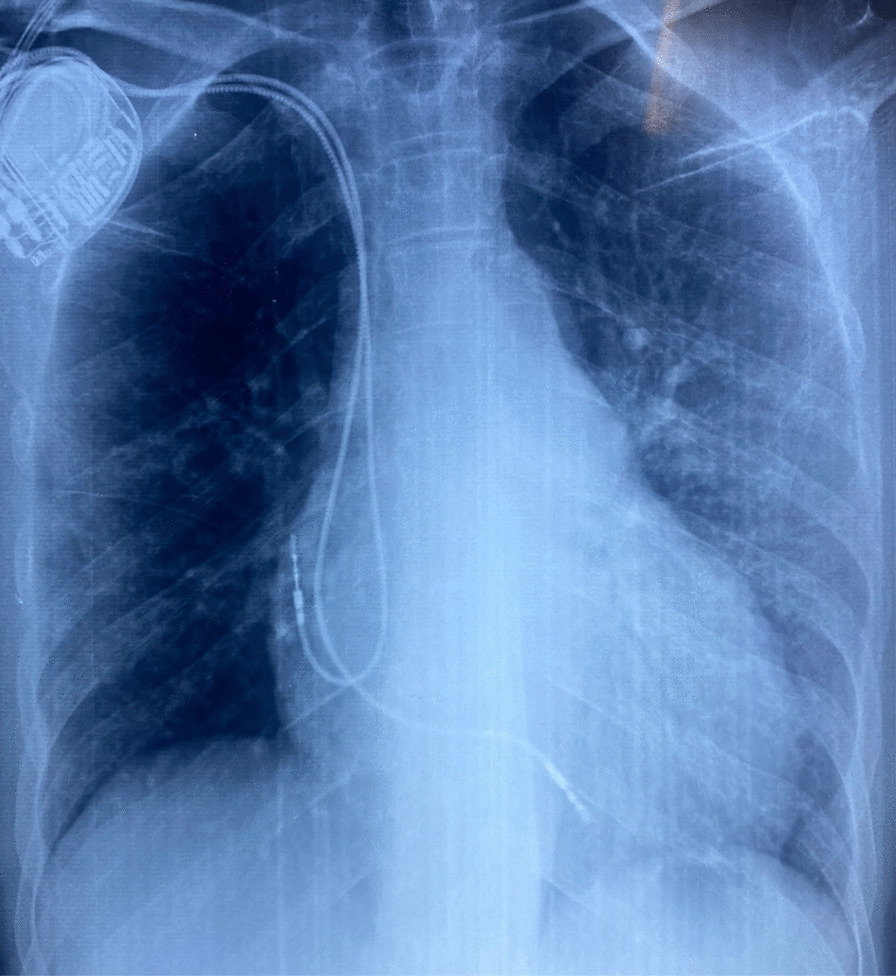
A transvenous dual-chamber permanent pacemaker implanted through the right subclavian vein

The first 12-lead electrocardiogram (ECG), showed a bifascicular block (Fig4A), combining a right bundle branch block (RBBB) and a left anterior hemiblock (LAHB). The second ECG performed 2 days later, showed a third-degree (complete) atrioventricular block (Fig. 4B). Transthoracic echocardiogram showed no signs of pulmonary artery hypertension, pericarditis, or endocardial or myocardial affections. Troponin and B-type natriuretic peptide (BNP) levels were within normal range, and the rest of the laboratory data were without particularities. On the basis of the clinical and electrographic data, the diagnosis of tachycardia–bradycardia syndrome was established. Therefore, a transvenous dual-chamber permanent magnetic resonance imaging (MRI)-compatible pacemaker was implanted through the right subclavian vein (Fig. 5), allowing the introduction of metoprolol. The patient no longer reported presyncope episodes or palpitations. She was discharged, and a cardiac MRI was scheduled to determine the extent of the fibrosis level.

## Discussion

SSc is considered a rare chronic multisystemic disease. It is characterized by the dysregulation of adaptive and innate immunity, microvascular damage, and skin and multiple internal organs progressive fibrosis [6]. Symptoms of conduction disorders and arrhythmias such as fatigue, palpitations, or syncope are frequently reported in patients suffering from SSc. According to the large European League against Rheumatism Scleroderma Trials and Research database (EUSTAR), arrhythmias and pericardial effusion represent the most frequent cardiac complications [7]. ECG abnormalities in SCC are common. Up to 75% of patients with SCC have abnormal ECG, and their presence is considered an independent predictor of mortality [8]. Among 128 SSc-related deaths reported in the EUSTAR database, 6% were attributable to arrhythmias [7]. A large study examining the noninvasive assessment of cardiac arrhythmias in 35 patients with scleroderma demonstrated that conduction defects were detected in only 19% of cases by ECG, essentially first- or second-degree atrioventricular block, and, unlike our case, not a complete atrioventricular block, whereas this percentage reached 33% with 24-hour Holter monitoring [9]. This further supports the use of 24-Holter ECG in this patient population when the context is suggestive of arrhythmia. In our case, the diagnosis of complete atrioventricular block was made using 12-lead ECG, whereas the diagnosis of tachycardia–bradycardia syndrome was made through Holter ECG. Nowadays, new noninvasive tools are used for myocardial subclinical dysfunction assessment. One of the most powerful is cardiac MRI [10], allowing the screening of late gadolinium enhancement (LGE) which is considered the most reliable method for myocardial fibrosis detection. In fact, an arrhythmia was diagnosed in almost 75% of patients with SSc with an LGE pattern [10]. Unfortunately, cardiac MRI remains a rather expensive tool, not easily available in our context. This can represent a real challenge, as early diagnosis of cardiac complications in SSc is crucial and allows early management of arrhythmia treatments. Anti-arrhythmic drugs should be used with extreme caution, because of their potentially harmful effects on other SSc organ disorders. For instance, beta-blockers are very effective arrhythmia treatments but, with the exception of certain new products, may aggravate Raynaud phenomenon in the other hand [8]. For this very reason, treatment with metoprolol was considered more appropriate in our case after pacemaker implantation.

## Conclusion

Arrhythmia and conduction disorders are by no means an uncommon complication of SSc. Early detection is key because they are associated not only with poor prognosis but also with a high risk of mortality. Despite the development of new noninvasive tests able to detect early cardiac involvement of SSc, management remains essentially symptomatic.

## Data Availability

Not applicable.

## Abbreviations

SSc: Systemic sclerosis
SSS: Sick sinus syndrome
ECG: Electrocardiogram
MRI: Magnetic resonance imaging

## Declarations

### Provenance and peer review

Not commissioned, externally peer-reviewed.
